# Growth

**Published:** 1891-11

**Authors:** 


### HALL'S
### JOURNAL OF HEALTH

TRUTH DEMANDS NO SACRIFICE; ERROR CAN MAKE NONE.

Vol. 38. NOVEMBER, 1891. No. 11.

### GROWTH.

If one were to suffer himself to read or listen to such doctrines and views only as accord with his preconceived notions, nothing would come of it to his advantage. There would be no inquiry or investigation into any thing new; no acquaintance with the progressive thought of the age. What a state of intellectual stagnation would be the outcome of such a policy, and yet this is just the condition of some people and assemblages of people who fail to perceive that it is only by the frictional contact of conflicting views that the truth is arrived at.

It is for this reason that we admit to our columns writers who present different sides of a question, leaving our readers to form their own conclusions as to their respective merits. Only rarely we receive a "Stop my paper" summons from some narrow-gauged individual, whose bigotry and self-conceit are a kind of disease which no medicine can cure. But there is consolation in knowing that in place of every such shallow-pated grumbler who would crush us by withholding his dollar, at least half a hundred new subscribers swell our subscription list, which, we are glad to say, has more than doubled the past twelve months.

Some people live in continual fear of having their settled convictions disturbed. They wouldn't for the world allow any thing, not even the truth, to come between them and their idols. This journal is far from being suited to this class of readers. Its habit is to instruct without flattery and condemn without malice.

We have, notwithstanding, a class of patrons which is never disturbed by conflicting opinions. Their concern is wholly outside of doctrines, precepts, and schools of medicine.

We allude to our advertisers, and they certainly have no reason to complain of our growing circulation. We wish them, one and all, great prosperity in their several enterprises, and they will have it, too, if the predictions of the JOURNAL are not amiss.

